# MOBBED: a computational data infrastructure for handling large collections of event-rich time series datasets in MATLAB

**DOI:** 10.3389/fninf.2013.00020

**Published:** 2013-10-10

**Authors:** Jeremy Cockfield, Kyungmin Su, Kay A. Robbins

**Affiliations:** Department of Computer Science, University of Texas at San AntonioSan Antonio, TX, USA

**Keywords:** MATLAB, database, time series, events, EEG, EEGLAB, big data, provenance

## Abstract

Experiments to monitor human brain activity during active behavior record a variety of modalities (e.g., EEG, eye tracking, motion capture, respiration monitoring) and capture a complex environmental context leading to large, event-rich time series datasets. The considerable variability of responses within and among subjects in more realistic behavioral scenarios requires experiments to assess many more subjects over longer periods of time. This explosion of data requires better computational infrastructure to more systematically explore and process these collections. MOBBED is a lightweight, easy-to-use, extensible toolkit that allows users to incorporate a computational database into their normal MATLAB workflow. Although capable of storing quite general types of annotated data, MOBBED is particularly oriented to multichannel time series such as EEG that have event streams overlaid with sensor data. MOBBED directly supports access to individual events, data frames, and time-stamped feature vectors, allowing users to ask questions such as what types of events or features co-occur under various experimental conditions. A database provides several advantages not available to users who process one dataset at a time from the local file system. In addition to archiving primary data in a central place to save space and avoid inconsistencies, such a database allows users to manage, search, and retrieve events across multiple datasets without reading the entire dataset. The database also provides infrastructure for handling more complex event patterns that include environmental and contextual conditions. The database can also be used as a cache for expensive intermediate results that are reused in such activities as cross-validation of machine learning algorithms. MOBBED is implemented over PostgreSQL, a widely used open source database, and is freely available under the GNU general public license at http://visual.cs.utsa.edu/mobbed. Source and issue reports for MOBBED are maintained at http://vislab.github.com/MobbedMatlab/

## Introduction

Traditional EEG research has focused on highly controlled experiments such as visual-oddball tasks performed in laboratory settings. Increasingly, researchers have recognized that the brain and body cannot be isolated from their environmental context and have started to measure active behavior using new monitoring and sensor technologies. The result is a dramatic explosion in the number, size, and complexity of datasets available for analysis. For example, a monitoring experiment to capture brain and body data during active behavior might record 256 channels of EEG along with eye tracking, motion capture, and respiration information for several hours at mixed sampling rates of 512 Hz or higher. Richer environmental contexts cannot be encoded as simple event streams of target presentation and user response. Rather, a mosaic of complex, interacting environmental conditions and events unfolds during the course of the experiment, leading to the idea of *event-rich time series*.

This explosion of data requires a computational infrastructure that allows ordinary users to more systematically explore and process collections of datasets. Basic tasks include managing, searching, and retrieving events across multiple datasets and handling complex event patterns containing environmental and contextual conditions applicable for a specified time interval (e.g., during this period of time, the subject is standing up and the lights are off). As datasets become larger, central archiving of primary data becomes necessary to save space and avoid inconsistencies. Systematic evaluation of algorithms and phenomena requires that primary data be subjected to consistent processing pipelines and that the output of these pipelines be cataloged and shared.

The goal of this work is to build a flexible database infrastructure that will support advanced large-scale analysis and data-mining applications for EEG and other types of sensor time-series within event-rich contexts. The remainder of the paper is organized as follows. We begin by introducing some motivating use cases and then describe the database organization of MOBBED and how it supports these use cases. We present examples of the MOBBED MATLAB programming interface and also provide performance measurements for several types of EEG collections. Finally we discuss some related work and offer some concluding remarks.

## Motivating use cases

The focus of most neuroscience databases is the storage and retrieval of datasets and the querying of experimental metadata. Extensible Neuroimaging Archive Toolkit (XNAT), for example, provides tools and database infrastructure for organizing and sharing data and metadata (Marcus et al., 2007). Such a database can serve as the replacement for the traditional laboratory notebook and has many advantages over its more traditional hardcopy counterparts by providing organizational structure, enabling data sharing, preventing information loss, and making information accessible for query.

Although the MOBBED provides some metadata storage capabilities, its purpose is quite different. The goal is to provide a lightweight, easy-to-use, extensible toolkit that users can incorporate into their computational workflow to store and query EEG and other types of data in an event-rich context. The remainder of this section introduces four motivating use cases for the development of the MOBBED toolkit.

### Creating and querying complex events and scenarios

Traditional EEG experiments have been conducted under rigidly controlled conditions using a small number of stimulus types (e.g., “red square displayed in right field of view”) and response types (e.g., “subject presses a button”). Downstream data analysis typically focuses on behavior around these time markers.

New technologies and research efforts such as the development of wireless EEG (Lun-De et al., 2012) and the ability to record complex task scenarios (Lance et al., 2011) result in significantly more complex stimuli, environmental context, and response scenarios. For example a military mission simulation might include video, audio, instrument status, and environmental feedback. Furthermore, these stimuli have many nuances identified as distinct event types. For example, in a situation where a subject is listening to audio communication, background communication is usually distinguished from communication targeted to the subject. Distinctions might also be made as to the importance of the communication, the relationship of the speaker to the subject, and whether the subject is expected to respond.

Events of this nature are generally “synthesized” by processing time-synchronized logs from external devices, and often researchers create different event overlays depending on the focus of the study. A database provides an ideal infrastructure for managing and querying extensive event overlays.

Event overlays, which can arise from many different sources, can be used to organize and evaluate the results of complementary analyses. For example, alpha spindles have been associated with degradations in driver performance (Simon et al., 2011). Researchers could apply an automated alpha spindle detection algorithm to driving simulator data to further explore this relationship. Each alpha spindle could be encoded as an event with a starting and ending time. If the detection algorithm provided a probability measure of correctness along with a classification label, the researcher might want to filter query results based on certainty of detection. The researchers could annotate the spindle event data with tags indicating that the events corresponded to alpha spindles detected during a driving simulation. Other information such as the location of the electrode used for the detection could be included as attributes of these events. Researchers could create a second event overlay with driver lane deviation markers derived from simulator recordings and another overlay containing markers derived from eye trackers indicating intervals when eye blink rate or other features were abnormal.

An appropriate database should allow researchers to represent this information in a way that will allow complex queries and exploration. With database support, researchers can specify and analyze complex event scenarios across multiple datasets and look for patterns. For example, researchers may want to distinguish and analyze the concordance of events of different types by asking questions such as:

Across a specified sub-collection of datasets, when did an event of type *A* occur within 500 *ms* of an event of type *B*? What other events occurred during that time?

In the driving simulator example, one could ask how often lane deviations occurred within 5 s of an alpha spindle.

### Mapping of event types across data collections

As researchers begin to tackle more complex questions about real-world behavior, larger data collections are needed to sufficiently cover the possible scenarios. Meta-analysis applied across multiple studies is increasingly appealing, both from the need for more data and to establish conclusions that are broadly applicable. A barrier to this analysis is the lack of a common vocabulary across datasets.

One way to circumvent vocabularies differences across laboratories is to allow users to associate arbitrary descriptive tags with events, much like Internet users assign tags to their shared images to enable searching. To assure overlap in tag vocabulary, Nima Bigdely-Shamlo and others (HED-homepage) have developed the Hierarchical Event Descriptor (HED) system, which is a hierarchy of standard descriptors for EEG and related experimental events. HED tags are of the form of path names (e.g., *Stimulus/Visual/Background/Uniform Color/Black* denotes a path from the root *Stimulus* → *Visual* → *Background* → *Uniform Color* → *Black*) and users can extend the hierarchy at the leaf nodes to enable experiment-specific tagging. Standard prefix processing can be used to match tagged events to a particular level of specificity.

Recently, several toolboxes have been developed to associate arbitrary lists of these tags with events either during data acquisition (Kothe, 2013a,b) or during processing (CTAGGER, 2013). To realize the potential of such a tagging scheme for data-mining, researchers need efficient tools for searching and matching tags across large collections of dataset events.

### Caching and reusing computations for efficiency and provenance

As data collections become more complex, it is harder to keep track of the output of different stages of the processing pipeline. Researchers usually preprocess raw data by applying various filtering transformations. They save selected intermediate results of these transformations on their local file system, often employing a naming scheme that reflects the processing. This approach has several drawbacks from a stability and reliability point of view. Often multiple copies of the files exist; these files only are accessible on the local machine; and there is no permanent documentation on the exact computations that produced the files. As a result, each time these files are needed, they are recomputed.

BCILAB (Delorme et al., 2011), a MATLAB package created by Christian Kothe as a platform for systematically developing and testing machine learning algorithms, has a caching system that provides a model for overcoming these issues. BCILAB represents each computation as a string containing the fully-parenthesized nested expressions back to the initial dataset and caches intermediate results locally so that they don't have to be recomputed during repetitive operations such as cross-validation. The cached results are hashed using the nested expression strings as keys. When performing an operation, BCILAB creates the string representation of the fully-parenthesized expression and successively parses the nested expression from outermost to innermost parentheses until it finds a string that appears in the hash table or it reaches the original dataset. BCILAB then evaluates the expression outward and stores and indexes the intermediate results if their computation time exceeds a threshold.

A database with the capability of mapping unique string identifiers to datasets can provide a third-level cache for systems such as BCILAB. The database provides more permanence and gives the user the opportunity to document the transformations in a standard way. The database server infrastructure allows users to access data from remote machines and to organize and control the sharing of their data and computations.

### Content-based EEG retrieval (CBER)

A motivating use-case in our own work has been the development of content-based EEG retrieval (CBER) systems (Su and Robbins, 2013). Content-based retrieval or query-by-example uses features from an example segment to retrieve similar segments from a database. Content-based image retrieval (CBIR) is well-established, with a variety of applications including enhancement of search in online image databases to automatic annotation and identification from surveillance video. As large scale EEG databases become available, CBER could enable a range of applications such as identification of similar subjects for enhancing brain computer interface (BCI) performance and discovering which environmental contexts are “enriched” when particular brain patterns occur.

## Database organization

We have built a MATLAB toolbox, MOBBED, that uses a PostgreSQL relational database to store datasets for access during computation. The toolkit includes a MATLAB layer for storing, searching, and retrieving data as well as an underlying Java layer that does not depend on MATLAB. This section provides a brief overview of the database infrastructure. Additional details on the database organization and information for programmers are available at (Cockfield et al., 2013a,b). MOBBED is designed for easy use in MATLAB workflows without any SQL programming. Readers who are not interested in the details of implementation may skip directly to the section describing the MATLAB interface.

### MOBBED dataset organization

**Figure 1:**
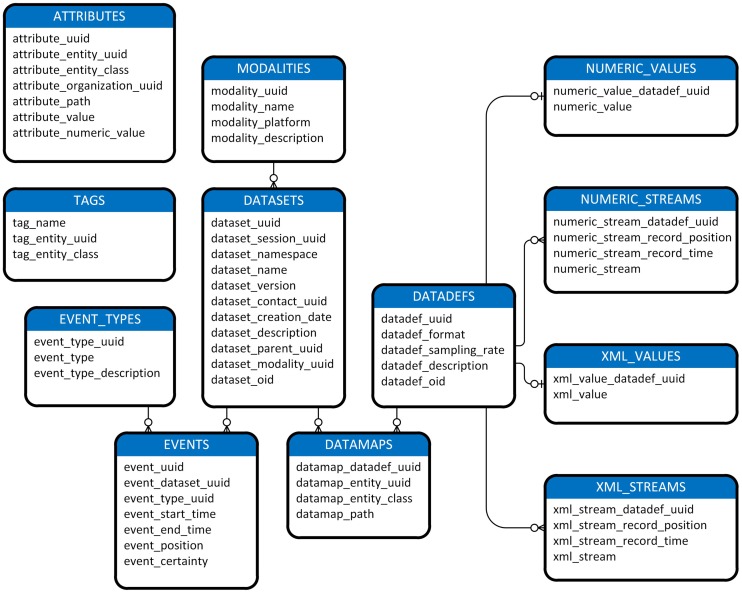
**A diagram of MOBBED data model**. The connecting lines indicate an association between rows of two database tables. An unmarked end always indicates exactly 1 of that item. The o-| combination indicates zero or one of the corresponding item. For example, the connection between DATADEFS and NUMERIC_VALUES indicates that each row in the DATADEFS table corresponds to either zero or one row in the NUMERIC_VALUES table, while each row in the NUMERIC_VALUES table corresponds to exactly one row in DATADEFS. The three prong connector specifies “many.” Tables containing an entity_uuid and entity_class column combination (i.e., ATTRIBUTES, DATAMAPS, and TAGS) may have associations with any table. For readability, these associations are omitted except for the connection between DATASETS and DATAMAPS, which is given as an exemplar.

Figure 1 shows a view of the MOBBED data model. Datasets are a central organizing concept for the MOBBED database. A dataset is a group of related items representing a single modality such as EEG usually collected from a single experimental run. Each dataset is uniquely identified by a 128-bit Universally Unique Identifier (UUID), which serves as the primary key for the DATASETS table. A session UUID allows scientists to group datasets that were acquired in the same session for easy retrieval. The dataset namespace allows investigators who share a database to avoid naming conflicts. Typically investigators use their individual laboratory URL as the namespace designator. The combination (dataset name, dataset namespace, dataset version) must always be unique.

Users can store and retrieve datasets from MATLAB using the high-level mat2db and db2mat methods. The mat2db always stores the actual dataset as a single large binary object, but may explode events and other metadata into the database to facilitate searching. The dataset modality indicates how a dataset is exploded into the database. Currently three modalities are supported: SIMPLE, EEG, and GENERIC, but other modalities can be added quite easily.

SIMPLE modality, which works for any type of data, just enters dataset information into the DATASETS table and stores the data as a large binary object. This modality is useful for archiving data. If the data is stored in some variable called mydata, the user simply creates a structure such as the following and calls the mat2db method.


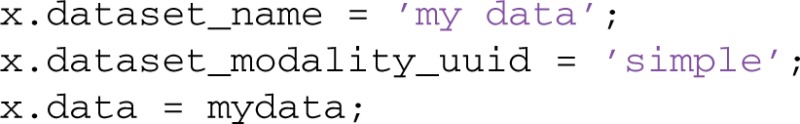


On retrieval using db2mat, the user will receive a structure such as the following, with the original data stored in the .data subfield. The remaining fields directly correspond to the columns of the DATASETS table. MOBBED fills in the fields that the user didn't provide with defaults.


x
  .dataset_uuid
  .dataset_session_uuid
  .dataset_namespace
  .dataset_name
  .dataset_version
  .dataset_contact_uuid
  .dataset_creation_date
  .dataset_description
  .dataset_parent_uuid
  .dataset_modality_uuid
  .dataset_oid
  .data


Although MOBBED doesn't explode events and other metadata in the database for SIMPLE modality datasets, users can still associate tags, attributes, and additional data with the dataset as described later.

EEG modality assumes that the dataset is a standard EEGLAB (Delorme et al., 2011) EEG structure and explodes the EEG.event, EEG.urevent, and EEG.chanlocs field values into the database. The GENERIC modality explodes existing x.element, x.event, x.feature, and x.metadata subfields into the database. As explained in the MOBBED user manual (Cockfield et al., 2013b), users can easily create an additional modality XXX by providing an XXX class with a store method and adding a row to the MODALITIES table using the MOBBED putdb method.

### Associating additional data with a primary dataset

Once a dataset (of any modality) has been created and has a UUID, users can associate arbitrary additional data with that dataset using data definitions. The DATADEFS table supports five formats of data: NUMERIC_VALUE (a vector of doubles), NUMERIC_STREAM (individual time-stamped vectors of doubles), XML_VALUE (an XML string), XML_STREAM (individual time-stamped vectors of XML strings), and EXTERNAL (an external data blob). If the data format is not EXTERNAL, MOBBED explodes the data into one of the data tables (NUMERIC_VALUES, NUMERIC_STREAMS, XML_VALUES, XML_STREAMS) so that it is available for searching. PostgreSQL supports vectors as data elements and can search within elements. If the data is a stream, sampled at a fixed rate, the sampling rate column of DATADEFS gives the sampling rate in Hz. Otherwise this entry is −1. The time stamps for stream data can always be recovered from the time-stamped stream itself.

The DATAMAPS table allows users to create an arbitrary number of associations of data definitions with datasets or other entities. For example, a researcher may wish to keep different Independent Component Analysis (ICA) decompositions of a dataset as auxiliary data using data definitions.

During a typical computational workflow, users may produce several versions of a dataset (e.g., after filtering, after artifact removal, etc.). MOBBED supports four methods of associating the new data with the original:

1. As a later version: If the IsUnique flag is set to false in mat2db, MOBBED automatically increments the dataset version when attempting to write datasets whose (name, namespace) already exists. If IsUnique is true (the default), attempts to perform such a write fail.
2. As child: Researchers often save multiple copies of a file at different stages of processing, appending monikers to the original file name to indicate processing steps. Following this strategy, a MOBBED user can set the parent UUID to the original dataset when using mat2db to store the new dataset with a modified name.
3. As a data definition: MOBBED users may also create data definitions for the additional versions and then add an association of the data definitions with the original through the data map.
4. As a transform: If the transformation of the original dataset to the new dataset can be represented as a string that can be evaluated to reproduce the new dataset, then this command string can be stored as a transform. These transform strings can be searched to determine whether a dataset derived by this transformation already exists and doesn't have to be recomputed (caching). If the transform string represents the original dataset by its UUID, forward searching can find all datasets that have been computed from an original dataset (provenance). Transforms are intended to be used in automated workflows.

### MOBBED event organization

Events provide contextual overlays for the data. Experimenters use events in traditional laboratory settings to encode the administered stimuli (e.g., target appears on right of screen) and user responses (e.g., button press). As experiments capture more realistic behavior in richer environments, the event overlays become more complex. Environmental conditions such as terrain or lighting changes, external noise, and motion may be encoded as events. Furthermore, data streams representing other modalities such as eye tracking, motion capture, or respiration monitoring might be processed to produce higher-level event streams (e.g., eye blink, saccade left, move left arm, respiration rate high). A central motivation for MOBBED is to support analysis of datasets that have complex combinations of events.

We define events as time-markers in the data that have a label (type) as well as a start time and an end time (which may be the same). Each event, which is identified by a UUID, has an entry in the EVENTS table. Events have start times and end times that are defined in seconds since the beginning of the associated dataset. Each event also has an event type, which is defined in the EVENT_TYPES table. Event types are strings identified by UUIDs. Researchers will typically reuse event types across multiple experiments to facilitate identification. Event can also have arbitrary data associated with them through the DATAMAPS table as well as attributes and tags, which are described in the next section. Event types are often tagged to facilitate searching across data collections.

The certainty value is specifically included as a column of the EVENTS table to provide an efficient filter when extracting events. Events can come from a variety of sources and increasingly these events will be the product of computation or synthesis of other data streams. Computational algorithms are not perfect and often these algorithms have an accuracy or probability associated with them. The event certainty is a value between 0 and 1 that captures this accuracy or certainty. The hardware-inserted markers of traditional EEG experiments will always have a certainty value of one, but results such as classification of audio streams into different types may not be as certain. Class labels output by classifiers can be defined as event types, and many machine learning algorithms produce certainty measures in various formats.

MOBBED automatically explodes events for EEG and GENERIC modality datasets. In each case the dataset is assumed to be in structure form with certain structure fields (.event for GENERIC and .event and .urevent for EEG). The .event.type or .urevent.type subfield is assumed to specify the event type. The storing of events is done automatically as part of storing the dataset. The event type, which is described in more detail in section on events, is important for identifying common events across datasets in data mining applications.

### MOBBED tags and attributes for storing metadata

A challenging aspect of complex event-rich time series is the variety of additional metadata that is possible. Specifying a fixed format for such data would make the system unusable for researchers. MOBBED takes a semi-structured approach to metadata using tags and attributes.

Tags, which are stored in the MOBBED TAGS table, are strings that can be assigned to any entity (dataset, event, data stream item, etc.) to facilitate searching. Tags can be ordinary strings or hierarchical path strings to support queries that match prefixes or use more general regular expressions. To enable mapping of events across different studies, the researcher can tag event-types or individual events. A rich and flexible vocabulary for tagging events is needed for effective data-mining across different data collections, particularly in real-world virtual or actual environments. For example, in a simulated driving study, the experimenters originally encoded an event corresponding to the subject (driver) passing a car as '[2, 2, 4]', which would make it impossible for an analyst to data mine without delving into the detailed experimental notes of the original investigator. However, if the original investigator tagged the data with information such as:


'/Time-locked Event/Stimulus/Visual/Complex/
 Dynamics/Passing/Same Direction/Overtaken
 by Subject'


The dataset could be tagged to provide a context for searching such as:


'/Context/Indoors/Simulator/Driving'
'/Context/Participants/Alone'


Hierarchical tag specifications allow users to select events or other entities at a specific level of detail, particularly if the original investigator tagged from a specified hierarchy. However, an investigator could have also used the individual tokens of these paths as the tags in a flat search scheme.

In contrast to tags, which are free-floating associations, attributes have a location in the entity structure, which is stored as a path. If the attribute path is '/event/target_dist', the value of the attribute was stored in x.event.target_dist of the corresponding data structure, x. The ATTRIBUTES table stores both the qualified entity's UUID and its class (table name) to facilitate searching. Although users can manually store attributes after creating a dataset to facilitate a particular search strategy, most attributes are stored at dataset creation time based on the modality definition. For example, MOBBED automatically maps channel locations and some event modifiers into attributes for EEG modality. Attributes can be either string or double. More complex data types can be encoded as XML strings or stored using the data definition mechanism in the appropriate format.

### Other MOBBED tables

MOBBED has several secondary tables, the most important of which are the COLLECTIONS and TRANSFORMS tables. A collection is an arbitrary grouping of entities. Collections can also be associated with attributes, tags, and data definitions. The TRANSFORMS table is essentially a hash table mapping provenance strings to data. Other tables include COMMENTS, CONTACTS, DEVICES, ELEMENTS, and SUBJECTS. Additional details about the database can be found in (Cockfield et al., 2013a).

## MATLAB interface

A simple, usable interface is the key that allows researchers to incorporate a computational database as an intrinsic part of their MATLAB scripting without learning SQL or becoming familiar with the underlying database schema. Careful thought has been given to default parameters to simplify scripting. The following section presents the MOBBED interface using examples that a typical researcher might use in practice. Additional details and complete interface specifications can be found in the MOBBED User Manual (Cockfield et al., 2013b).

Table 1 summarizes the MATLAB interface for MOBBED. The functionality is encapsulated in a MATLAB class, Mobbed, which also has static methods to create and delete databases. To access a database, the users create a Mobbed object and call methods on this object. For users unfamiliar with MATLAB object syntax, method calls are similar to ordinary function calls except that the first argument is the variable containing the object. Each of the methods listed in Table 1 is treated as a single transaction. That is, all of the operations needed to complete the call are either performed successfully or the operations are undone (rolled back) so that the underlying database is unchanged.

**Table 1:** **MATLAB-database interface (Mobbed MATLAB class)**.

**Method**	**Description**
mat2db	create and store a dataset in the database
data2db	create a new data definition and store corresponding data in the database
db2mat	retrieve a dataset from the database
db2data	retrieve a data definition from the database
getdb	retrieve rows from a single table
putdb	create or update rows from a single table
close	disconnect from the database (i.e., further calls cause an exception)

The mat2db method stores a dataset in the database, and the db2mat method retrieves a dataset from the database. The getdb method retrieves rows from any database table using simple, but flexible search criteria. The putdb method creates or updates individual rows in a specified MOBBED database table and is used to add tags, attributes, or event overlays after storing the dataset with mat2db. The close method disconnects from the database and releases associated resources.

The remainder of this section presents some examples using the Mobbed methods, assuming the original datasets are stored in EEGLAB EEG structures. Runnable MATLAB scripts containing these examples as well the examples in the User Manual come with the MOBBED distribution. The examples assume that the user has installed PostgreSQL on the machine that will host the database.

### Creating and deleting a database

The following example creates a database on the local machine using the createdb static method of Mobbed: 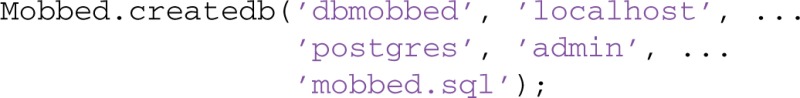


The first argument is the name of the database, and the second argument is the name of the machine hosting the database (in this case the local machine). To create a database on a different machine, replace 'localhost' with the IP address or hostname of the host machine. This example uses the default 'postgres' user with a password of 'admin' to create a database called 'dbmobbed'. The mobbed.sql script, which comes with the distribution, contains the SQL code needed to create the database. The createdb function throws an exception if an error occurs.

Once created, a database has permanent existence until it is deleted, independently of whether MATLAB is running. Users can use the pgAdmin tools (pgAdmin-homepage) or web-based tools to examine the data outside of MATLAB.

The deletedb method of Mobbed deletes a particular database: 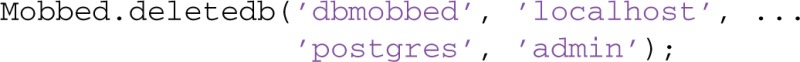


Users should exercise caution in using deletedb, particularly if the database is being shared among users.

### Accessing the database in MATLAB

Within MATLAB, users access a database by creating a Mobbed object connecting MATLAB to the database and calling the methods listed in Table 1 using a handle for this object. The following statement creates a connection to the 'dbmobbed' database that resides on the local machine: 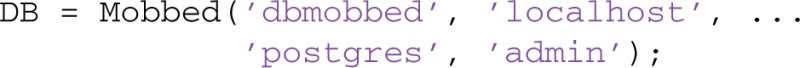


DB is the connector object handle used for future references using this connection. Users may open connectors to multiple databases or have multiple connections to the same database active at the same time. To close a connection use:


close (DB);


### Storing datasets in the database

Users can store and retrieve datasets using the mat2db and db2mat methods, respectively. MOBBED uses structures to pass information to and from the database, so users don't have to know SQL or be aware of details of the underlying database organization. The following example loads a previously saved EEGLAB EEG structure into MATLAB and then writes the result to the database: 
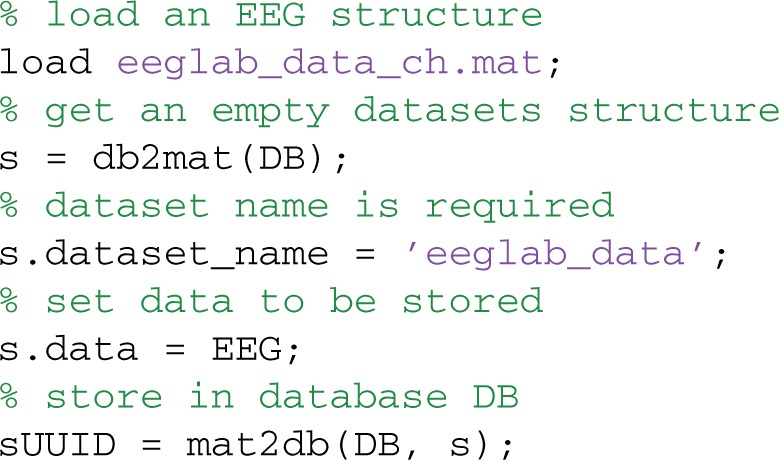


To store a dataset, first get an empty dataset structure from the database by calling db2mat. The returned structure, s, is a template to be filled in. The only field that is required to be a given value is s.dataset_name. The s.data field should contain the actual dataset data, which is stored as a large binary object for easy retrieval. Store the dataset in the database by simply calling mat2db with the filled in structure. The example file comes with the MOBBED distribution.

The mat2db method returns a string containing the UUID that identifies the dataset in the database. The system uses 128-bit UUIDs for all keys, which allows records from multiple sources to be manipulated and merged without conflict. Since the example sets only the name and data fields of the input structure, s, MOBBED uses the default contact ('System'), the default namespace ('mobbed'), the default parent UUID (‘591df7dd-ce3e-47f8-bea5-6a632c6fcccb’), and the default modality ('EEG'). MOBBED not only stores the actual data as an external file, but also explodes the dataset events and channel information into the database to facilitate searching. The next section describes commands for exploding additional data into the database.

The mat2db method also allows additional optional parameters. For example, the following call associates the tags 'EyeTrack', 'VisualTarget', and 'AudioLeft' with the dataset: 
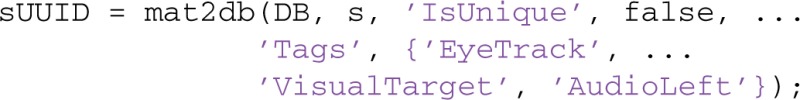


Since the keyword-value parameter 'IsUnique' is false, MOBBED increments the version if a dataset already exists with that namespace and name combination. On return, sUUID contains the UUID of the newly created dataset.

### Searching for and retrieving datasets from the database

To provide an interface that is MATLAB-like, MOBBED uses structures that mirror database tables. The getdb provides a flexible SQL-free query mechanism for searching by returning an array of structures with specified rows of a particular database table:


mStruct = getdb(DB, table, limit, varargin)


The limit argument specifies the maximum number of rows to return. The following statement retrieves a structure array containing all of rows in the DATASETS table: 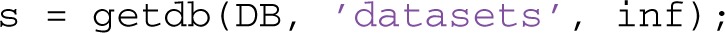


Users may qualify the search by specifying particular table column values as well as tag and attribute values. The getdb method only returns row-sets of the specified table, which includes metadata of various types, but no actual data. The next example uses getdb to retrieve up to 10 dataset rows matching a qualified search: 
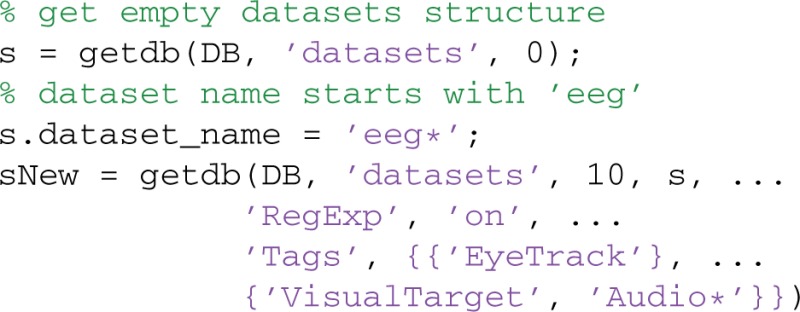


The first call to getdb retrieves an empty structure to be filled with information specifying a qualified search. The second call to getdb retrieves DATASETS rows based on four types of qualifications. The first qualification limits how many matches are retrieved as specified by the third argument of getdb, with inf indicating all rows and 0 indicating the return of the empty row structure for the specified table.

The fieldnames of the structure, s, returned by the first call have a one-to-one correspondence to the DATASETS table column names. The second qualification allows users restrict the retrieved column values by setting match criteria in the fields of s.

String columns may be queried in one of three ways: by direct match, with a cell array of allowed choices, or by matching a regular expression. For example, the qualification s.dataset_namespace = 'www.cs.utsa.edu' restricts the search to datasets whose namespace is 'www.cs.utsa.edu'. The qualification s.dataset_namespace = {'www.cs.utsa.edu', 'restricted'} restricts the search to datasets whose namespace is either 'www.cs.utsa.edu' or 'restricted'. If 'RegExp' is 'on' as in the above example, the choices are interpreted as regular expressions rather than direct matches. The qualification s.dataset_name = 'eeg*' specifies datasets whose name starts with the string 'eeg'.

Structure fields corresponding to database table columns containing UUIDs allow a single UUID value query or a cell array of UUIDs. Numerical columns allow query by matching particular numeric values.

The third qualification uses combinations of AND, OR, and regular expressions with tags. The getdb command allows an arbitrary number of tag groups, specified by a keyword-value pair. The keyword is 'Tags' and the value is a cell array of tag groups. Each tag group is a string specifying a single tag or a cell array of string tags. Every tag group must be matched for the item to be retrieved (AND). Within a tag group, at least one item must match the query for the tag group to be matched (OR). In the example, the dataset must have an 'EyeTrack' tag and either a 'VisualTarget' tag or a tag that starts with 'Audio'. The specification: 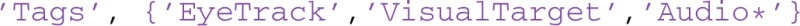 has three separate tag groups, and the datasets must match all three tags. Matching of individual tags supports regular expressions if 'RegExp' is 'on'.

The fourth qualification matches attributes instead of tags. Attributes are similar to tags except that attributes are structured, meaning that they have a particular position in the data structure relative to the item they qualify.

The retrieved datasets must match at least one value in each tag or attribute group as well as the qualifications on the column values. The UUIDs returned in the sNew structure array provide the keys for retrieving the complete datasets using db2mat: 
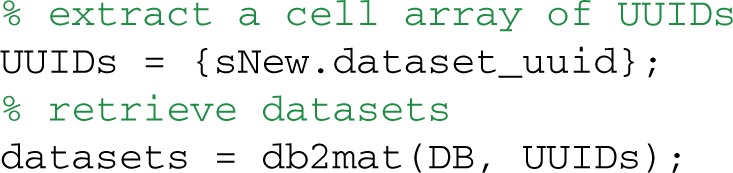


The returned datasets structure array has fields corresponding to the DATASETS table column names, plus a data field containing the actual data (in this case an EEGLAB EEG structure):


EEG = datasets(1).data;


The getdb method also supports data cursors for iteratively fetching the results of a query. In the following example, we process 100 events at a time corresponding to the datasets identified by UUIDs: 
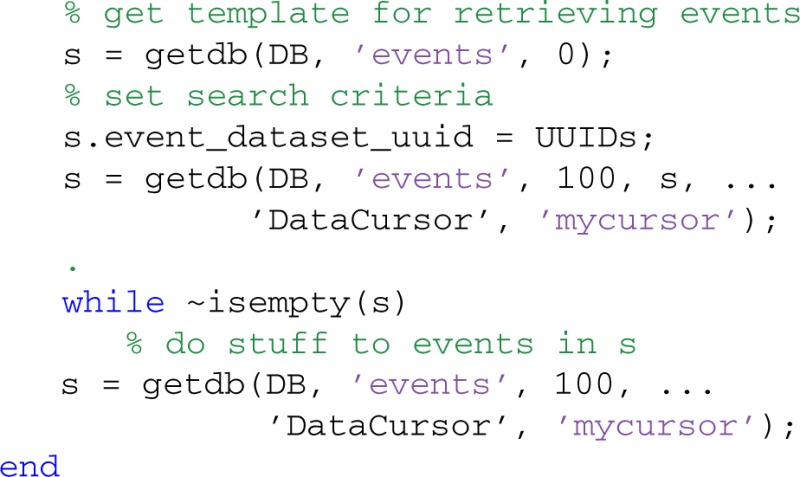


The first getdb gets the EVENTS table template to fill in, while the second getdb initializes a data cursor called 'mycursor' and retrieves up to 100 events corresponding to datasets whose UUIDs are in the cell array UUIDs. The getdb in the loop fetches the next 100 events after processing the previous set. MOBBED supports data cursors only for the getdb method. Single datasets are always retrieved in their entirety from the externally stored binary object.

### Events

A key to manipulating large-scale event-rich environments is the correct association and tagging of events across multiple datasets. Events in different datasets may have the same names, but completely different meanings, even within the same laboratory. Tags are a valuable step in this process. While the meaning of *“ButtonPress”* may vary dramatically from study to study, *“ButtonPress”* tagged events are likely to be more similar to each other than say *“ButtonPress”* is to *“TargetAppears.”*

However, tags are not the complete solution. In order to give researchers finer grain control over event associations across multiple datasets and studies, MOBBED supports event types that are defined uniquely by UUIDs. These types play the role of the “event codes” that researchers typically assign during data acquisition. Usually a researcher runs experiments over multiple subjects and conditions using a common set of event codes. If the researcher anticipates combining multiple studies for analysis, he or she may reuse these codes or map the correspondence between the codes across the studies. The MOBBED event types are designed to support this process.

When a user stores a dataset using mat2db, MOBBED automatically creates a unique event type for each unique value of EEG.event.type. (The dataset modality, in this case EEG, determines the particular name of the field.) The mat2db method returns a list of these unique types, which the user can then pass to successive calls to mat2db to reuse the same codes. During analysis, an analyst can extract events of a particular event type and be assured that the experimenter believed that these events were the “same.” The event types also provide a convenient way for researchers to provide detailed annotation, making it easier for others to understand what the type or code represents.

The following example illustrates how a user can create a set of event types that is consistent over multiple datasets using the 'EventTypes' optional argument of mat2db. This optional argument specifies the event types corresponding to the names of events in the experiments. MOBBED creates a new event type for each event whose type or code does not correspond to an event type already in this list: 
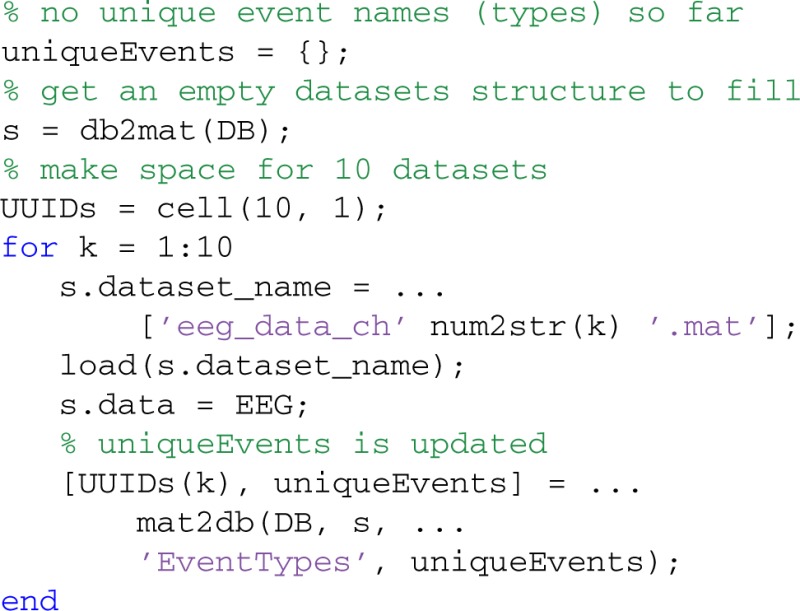


The example loads ten files: eeg_data_ch1.mat,…, eeg_data_ch10.mat and stores each dataset in the database. Each data file contains an EEGLAB EEG structure. Initially, the uniqueEvents structure is empty, and MOBBED creates a unique set of event types when storing the first dataset. For EEG datasets, the values in EEG.event.type are converted to strings and used as the event type. The 'EventTypes' value is a list of EVENT_TYPE_UUID keys for the EVENT_TYPES table. If the type string matches the EVENT_TYPE value corresponding to any of these keys, then MOBBED uses the corresponding key as the EVENT_TYPE_UUID. If MOBBED doesn't find a match, it creates a new type and adds the UUID to the uniqueEvents set. When the loop completes, uniqueEvents contains a complete set of event types for this data collection, having reused event types where appropriate.

### Storing and retrieving additional data

MOBBED supports several methods of associating additional data with a dataset: by versioning, by parentage, by creating a data definition, or by creating a transformation. The data definition approach is particularly useful for associating secondary data or feature vectors with the data. The additional data can be stored as a large data blob or can be exploded into the database in various formats to allow search and retrieval of individual pieces. Transforms are discussed in the section on caching, reuse, and standardization.

The following example illustrates MOBBED data definitions. Suppose a user wanted to store individual frames of the sample EEG dataset used in the previous section. The following code creates a data definition that stores each data frame of the dataset individually with a timestamp: 
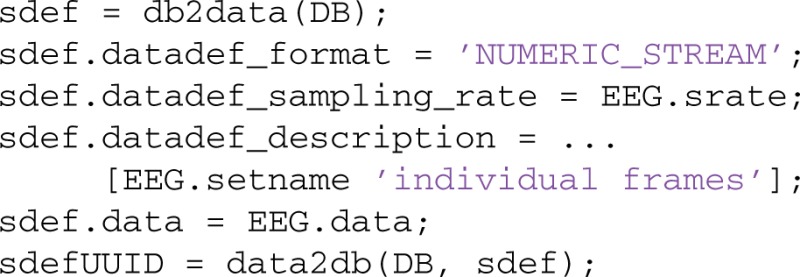


The call to data2db stores the data in a numeric stream format (each column of the data field is stored individually for direct access). The individual columns have time stamps that give the time in seconds relative to the start of the data, with spacing of the reciprocal of the sampling rate. Other formats include large data blobs (external), xml streams, numeric, and xml. The numeric can be a single value or a vector of arbitrary size. For example, to store epoched data for direct retrieval, simply provide an array with the epochs in the columns as data and a sampling rate of 1. Alternatively you can provide timestamps of the epoch starts instead of the sampling rate. Windowed power spectra can be stored in this way or externally as large data blobs. PostgreSQL incorporates some machine learning algorithms such as K-Means clustering through the MADLIB library (Hellerstein et al., 2012). Individual features can be stored in a data definition and clustered using calls to this library.

The data2db method stores data in MOBBED, but does not associate it with any datasets or other entities. To associate a data definition with a particular entity such as a dataset, you create a data map entry for the association. The following code segment maps this data to all 10 of the datasets loaded in the previous section. The structure path indicates how this data is associated with the entity. In the example, the data should be mapped to the .dataEx field of the dataset: 
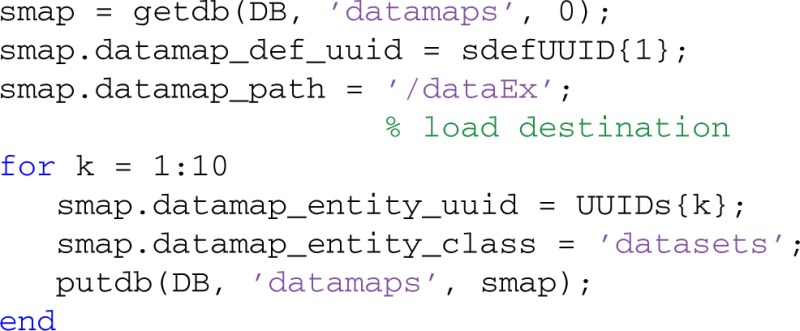


The db2data method returns the reconstituted data given the data definition UUID: 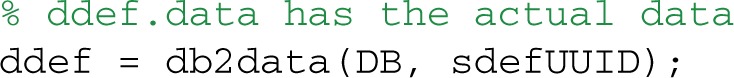


More typically, a user will want to retrieve all of the data associated with a particular dataset. The db2data method has an alternative form in which the rowsets from the data maps are passed as an argument. The following code segment retrieves all of the data items associated with the dataset whose UUID is in UUIDs{1} and puts them in the structures specified by the data map. In the example shown below, the dataset has one data item, which will be returned in the ddef.data.dataEx structure variable: 
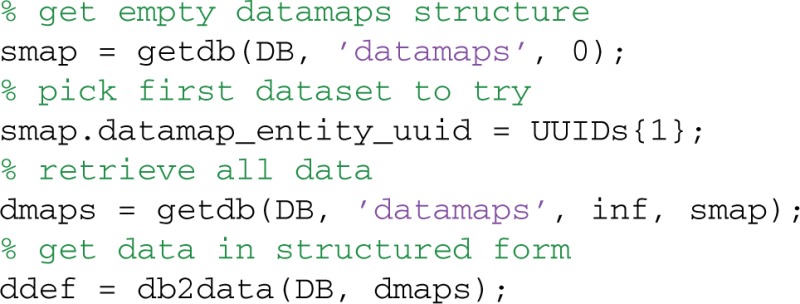


When presented with multiple row sets, db2data returns the result for the ith row set in ddef.data(i).dataEx.

### Caching, reuse, and standardization

MOBBED can also be used for caching expensive results and facilitating the reuse of calculations produced by standardized pipelines. Many groups have a standardized automated pipeline for processing their data and use a file naming convention to designate the results. Such approaches usually produce multiple copies of the processed data, and investigators can easily lose track of these copies. Sharing among group members or across groups compounds these difficulties. Often each individual will end up redoing the processing at great computational expense to be certain of the starting point and processing steps.

Many workflow-oriented programs and packages partially track the processing history or provenance of the data to improve reproducibility, and a number of journals and conferences encourage or require that authors make their code and data available after paper acceptance. Unfortunately, most users have not adopted tools to facilitate provenance, and no standards have emerged for doing so. True provenance (which requires every bit of history to be saved) can be quite difficult, making histories unreadable and unusable for ordinary users (Chapman and Jagadish, 2010).

MOBBED provides a simple provenance mechanism that users can incorporate into their workflows to facilitate caching, reuse, and standardization. The steps are as follows:

1. Store the original data in the database and obtain its UUID (mat2db).
2. Apply the processing pipeline to the data to obtain a new dataset.
3. Store the resulting data in the database and obtain its UUID (mat2db).
4. Choose a transform string that unambiguously identifies the transformed data.
5. Add the UUID and transform string to the TRANSFORMS table (putdb).

Suppose an original dataset has been uploaded to the database from the 'eeglab_data_ch.mat' data file and that the resulting UUID is available in sUUID{1} (step 1).

For illustration purposes, assume that the pipeline consists of calling the EEGLAB pop_eegfilt command to high-pass the data at 1 Hz (step 2):


EEG = pop_eegfilt(EEG, 1.0, 0, [], 0);


After we execute the following code to save the EEG dataset in MOBBED, the sUUIDNew{1} field contains the resulting UUID (step 3): 
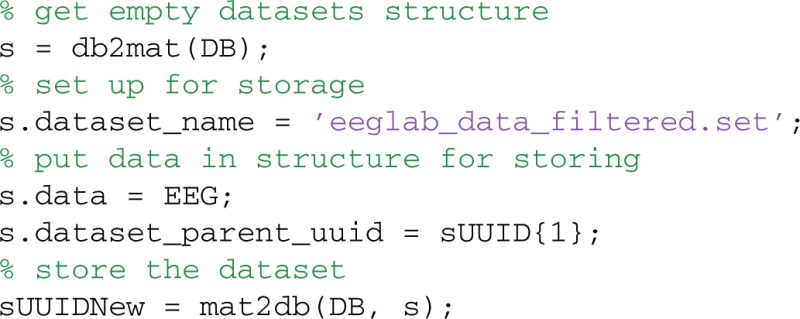


The user can choose any transform string, but the choice should be something that unambiguously identifies the result and can be computed or remembered. An example string might be constructed as (step 4): 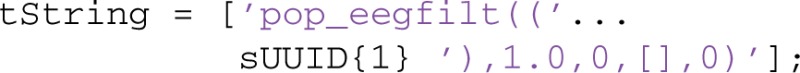


The tString transform string can be created from the original command by substituting the UUID corresponding to the input EEG structure for the EEG argument and removing extra blanks. The EEGLAB pop functions produce a suitable string for transformation in the command history.

The result can then be stored as a transformation for later lookup (step 5): 
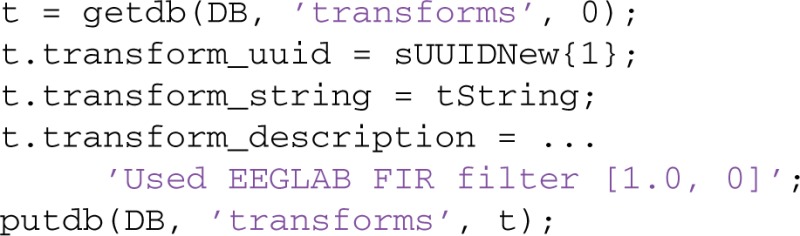


Once the transform is in the database, researchers can retrieve the result using getdb rather than recomputing it. The following example retrieves the row(s) of the TRANSFORMS table using the transform string: 
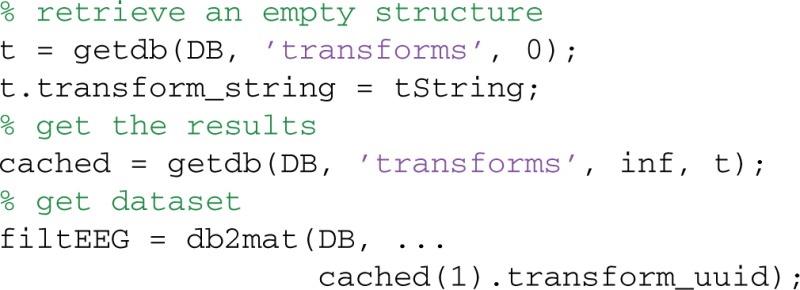


This strategy promotes sharing and organizes reuse more systematically than a simple file naming convention and documents the process in as much detail as the user chooses. To make this transformation process reliable, users would need to develop processing scripts that used wrapper functions to include database logging automatically.

### Threading and parallel processing

MOBBED also supports parallel processing and multi-threading if the user has the MATLAB Parallel Computing Toolbox as illustrated by the following example. The threads variable contains the number of workers used for parallel computing. For local processing, this should be less than the number of cores the desktop has. The fUUIDs variable is a cell array containing lists of UUIDs of datasets to be processed by each thread or worker: 
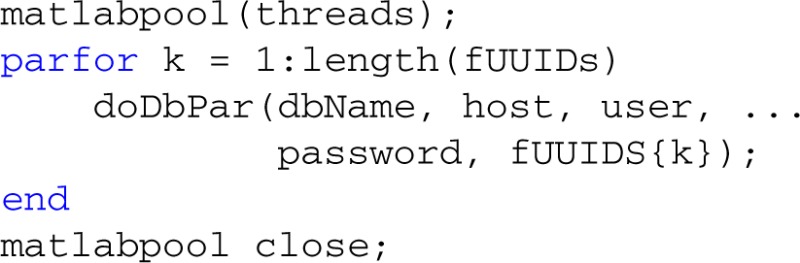


An example of the doDbPar is shown below. The key to using MOBBED in parallel processing is to open a connection to the database within each worker itself rather than passing an open connection as an argument, since all arguments to the worker functions must be serializable: 
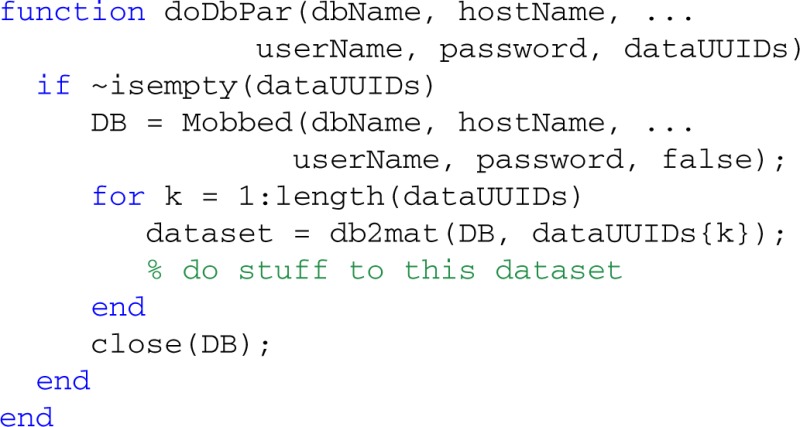


The distribution provides a number of example functions that illustrate various uses of parallel processing.

### Implementation

An overview of the MOBBED tables is shown in Figure 1. The getdb and putdb methods provide a 1-to-1 mapping between database columns and MATLAB structure fields. These methods determine the fields and their types directly by interrogating the database. The getdb method allows regular expressions and other qualifications for the supported types: char varying, double, bigint, and UUID.

It would be possible to use the reflection facilities of JAVA and MATLAB to map fairly general structures into the database. We decided against this approach for a number of reasons, most especially efficiency and complexity. Instead we use the concept of a dataset modality to define how a particular MATLAB dataset should be exploded into the database. Many tools represent their datasets using some type of standardized format. For formats other than the supported ones, the user would need to implement a new modality using EEG_Modality as a model. Once this is accomplished, search and retrieval is performed as before. Any dataset can be stored using the SIMPLE modality, which creates an entry in the DATASETS table and stores the tags or attributes associated with the dataset itself. MOBBED also stores every dataset as a large binary object retrievable by db2mat, irrespective of modality.

The mat2db and data2db methods each require a structure whose fields reflect the columns of the DATASETS and the DATADEFS table, respectively. To use these methods, users retrieve an empty structure and fill in the fields, leaving default entries blank. In each case, the user must set the .data field of the structure to the corresponding data before calling the method. The mat2db stores a dataset, while the data2db creates a data definition and stores auxiliary data in the specified format. Storing auxiliary data is generally a two-stage process—the user first creates a data definition with data2db and then uses putdb to create any number of entries in the DATAMAPS table associating this data item with other entities in the database. Any MOBBED entity can have associated data through this mechanism.

The CTAGGER tools for systematic tagging of event streams have recently been released (CTAGGER, 2013). These tools support efficient tagging of events represented in MATLAB through the .event subfield in a format that is consistent with the EEG modality (as used by the EEGLAB EEG structure) as well as the GENERIC modality. The CTAGGER tools store the tags in a standardized format in the .etc.tags subfield. The next release of MOBBED will automatically read this standardized format and store the tags for both of these modalities.

The MOBBED User Guide (Cockfield et al., 2013b) provides additional details and many more examples of the mapping between MATLAB structures and the database.

## Performance

In order to assess the performance and overhead of adding a database to the MATLAB workflow, we tested basic database operations on four different data collections summarized in Table 2.

**Table 2:** **Attributes of test datasets (average number per dataset)**.

**Data collection**	**Datasets**	**Subjects**	**Channels**	**Frames**	**Events (unique)**	**Event attributes**
EEGLAB (Delorme and Makeig, 2004)	10	1	32	30,504	154 (2)	4
Attention (Onton et al., 2006)	40	39	36	654,234	5,571 (17)	4
Shooter (Kerick et al., 2007)	112	14	40	206,487	270 (17)	18
BCI2000 (Schalk et al., 2004)	1526	109	64	18,226	26 (16)	3

The first collection consists of the small, relatively simple dataset distributed with EEGLAB that is used for the examples in this paper. We created 10 identical copies of this dataset to form a collection. Each dataset has 154 different events, but the events are only of two distinct types.

The second collection (attention) contains large datasets, each having a large number of events. The third collection (shooter) contains large datasets, each with a moderate number of events. However, the events have a large number of attributes that must be stored individually for each event. The fourth data collection (BCI2000) is publicly available and contains a large number of datasets. The individual datasets were stored as EEGLAB EEG structures in .mat files on the local files system for all tests.

To test the performance, we used three identical machines whose specifications are shown in Table 3 (one running the Windows 7 operating system and two running Linux). We did a clean install of the operating system and software on the three machines. No other programs were installed. We ran eight different configurations. The local configurations refer to MATLAB and the database running on the same machine. Remote configurations refer to configurations in which the database resided on a Linux machine separate from the client machine, but on the same sub network. We also ran parallel versions of the tests using two connections to the database and the MATLAB parfor command.

**Table 3:** **Configuration of test machines for performance tests**.

**Item**	**Description**
Processor	Intel Core i5-2400 quad-core processor 3.10 GHz, 1 MB L2 + 6 MB L3 cache
Memory	16 GB DDR3-1333 MHz SDRAM
Disk	Hitachi Deskstar 3 TB 7200 RPM SATA 6.0 Gb/s 3.5-Inch
OS	Windows 7 Professional or Ubuntu 12.04 LTS
MATLAB	2012a
PostgreSQL	Version 9.2

Table 4 shows the timing results for basic operations on the four different data collections of Table 2. Each entry represents the time in seconds averaged over a dataset. Values in parentheses indicate the parallel version with two threads.

**Table 4:** **MOBBED performance in time in seconds averaged over the datasets within each collection**.

**Description**	**EEGLAB**	**Attention**	**Shooter**	**BCI2000**
**LOAD/STORE LOCAL.mat FILE:**				
Windows	0.15 (0.08)	4.42 (3.31)	1.44 (0.96)	0.41 (0.23)
Linux	0.15 (0.07)	4.45 (2.72)	1.42 (0.84)	0.41 (0.22)
**STORE DATASET IN db:**				
Windows	0.67 (0.44)	14.50 (9.42)	5.36 (3.88)	0.62 (0.53)
Linux	0.77 (0.47)	14.66 (8.83)	6.73 (4.16)	1.25 (1.06)
Linux-Linux	0.87 (0.54)	16.08 (9.60)	7.21 (4.53)	1.15 (1.02)
**RETRIEVE DATASET FROM db:**				
Windows	0.15 (0.11)	1.87 (1.03)	0.60 (0.32)	0.08 (0.04)
Linux	0.18 (0.10)	1.99 (1.12)	0.70 (0.31)	0.10 (0.04)
Linux-Linux	0.22 (0.14)	2.90 (1.67)	1.05 (0.56)	0.12 (0.06)
**CREATE A db DATA DEFINITION FOR FRAMES:**				
Windows	1.74 (1.58)	37.97 (33.34)	14.85 (12.36)	2.92 (2.90)
Linux	1.63 (1.22)	40.46 (34.36)	17.35 (14.55)	3.19 (3.35)
Linux-Linux	1.72 (1.27)	44.29 (34.46)	17.71 (13.39)	3.64 (3.36)
**RETRIEVE FRAMES FROM db DATA DEFINITION:**				
Windows	4.61 (2.34)	77.73 (42.34)	25.28 (13.02)	2.54 (1.29)
Linux	4.83 (2.45)	83.35 (44.00)	27.10 (13.79)	2.87 (1.40)
Linux-Linux	4.93 (2.43)	85.44 (43.56)	28.24 (14.37)	2.98 (1.48)
**RETRIEVE EVENTS FROM db:**				
Windows	0.05 (0.09)	0.32 (0.32)	0.02 (0.02)	0.002 (0.002)
Linux	0.05 (0.09)	0.25 (0.27)	0.02 (0.03)	0.002 (0.002)
Linux-Linux	0.05 (0.09)	0.26 (0.28)	0.02 (0.02)	0.002 (0.002)

*Values in parentheses are for the parallel version with two workers*.

The first section of Table 4 gives the average time in seconds to load a dataset (from a .mat file) and store it as a temporary file on the local file system. A load and store operation is performed each time a dataset is uploaded to the database in the current implementation, so these values indicate how important this overhead is in overall performance.

The second section of Table 4 provides the average time to store each dataset in the database. The events are exploded into the database, including event attributes and channel information. The entire dataset is stored in the database as a large binary object external to the database tables. The times shown are averaged over each dataset, so datasets with many events and time points, such as the attention, take longer.

Ordinarily, datasets are retrieved from the database using their representation as large binary objects. The third section of Table 4 shows that retrieval from the database can be comparable to reading and writing to the local file system.

The fourth section of Table 4 shows the average time in seconds to explode the vector time samples of the dataset as individual searchable items in the data table. Again, since these values represent the average for a dataset, collections containing long recordings (e.g., attention and shooter) take longer. The next section shows the time it takes to reassemble individual frames into dataset from the exploded data.

The final section shows the average time per dataset to retrieve all of the events of the dataset without retrieving the entire dataset. The shooter data has complex event attributes and the attention collection has a significant number of events per dataset, so the average time per dataset is significantly longer for these. PostgreSQL has a vacuum operation, which should be run after a large number of writes or modifications. Each database was vacuumed between the storage operations and the retrieval.

The process was also run with two workers (and four workers—not shown) using the MATLAB Parallel Processing Toolbox. The performance improved by a factor of approximately 1.7 for each doubling of the number of workers. The benefit of multithreading is most apparent for large retrieval operations, and a nearly linear speedup has been observed for the individual frame retrieval from independent datasets.

## Related work

Data sharing and integration of searching and retrieval of databases with computational analysis is common in bioinformatics (Galperin and Fernández-Suárez, 2011) and has resulted in an explosion of large-scale reproducible analysis. In contrast, the sharing of neurophysiological data and tools is in its infancy (Akil et al., 2011; Koch and Reid, 2012; Poline et al., 2012), and systematic integration of EEG data into computational pipelines is uncommon. The number of publicly available recordings of human brain activity such as EEG and ECOG is also very limited. Two large EEG collections are available through PhysioNet (Goldberger et al., 2000): a 109-subject data collection of multichannel EEG recordings of BCI motor tasks (Schalk et al., 2004) and a 22-subject data collection of multichannel EEG recordings of pediatric seizure patients (Shoeb, 2009). Schulze-Bonhage et al. (2011) demonstrate the importance of large-scale high-quality datasets in their retrospective comparison of methods for predicting seizures from EEG.

Clearinghouse-type resources such as the Neuroscience Information Framework (NIF) (Gardner et al., 2008; Bandrowski et al., 2012), the International Neuroinformatics Coordinating Facility (INCF) (De Schutter, 2009), and Neuroimaging Informatics Tools and Resources Clearinghouse (NITRC) (Luo et al., 2009) provide portals to heterogeneous collections of neuroscience resources, but do not provide tools for integrating these resources into computations. The EPILEPSIAE (Evolving Platform for Improving the Living Expectations in Subjects Suffering from Ictal Events) project is a European effort to produce a high-quality, highly-annotated database of multi-channel continuous recordings of 300 subjects that includes EEG (Ihle et al., 2012).

Databases are rarely incorporated into MATLAB time-series workflows in general and EEG research in particular. The semi-structured nature of the data makes it difficult to map this data into a traditional relational database, and most users would prefer to avoid dealing with schemas and SQL queries in their workflows. G-Node (Herz et al., 2008) offers an online database for users to archive their data in a platform neutral manner. They offer a MATLAB client that allows users to import datasets and associated metadata into MATLAB and to write information back to the database. G-Node allows users to maintain a parent-child relationship among datasets and to store metadata along with experimental data (Grewe et al., 2011). G-Node is meant to provide archival storage and high-level provenance for data-collections, while MOBBED is meant to directly support large-scale computation in event-rich environments. The two database approaches are complementary, and G-Node structures can be mapped to and from a MOBBED representation in a straightforward manner, although MOBBED currently does not provide utility functions to do so.

A few MATLAB software packages keep internal data structures to organize information across multiple datasets. EEGLAB (Delorme and Makeig, 2004; Delorme et al., 2011), a general purpose MATLAB-based platform for EEG and MEG, has a STUDY structure that allows users to group a collection of datasets to perform a statistical comparison across subjects and conditions. Brainstorm (Tadel et al., 2011) also supports data structures for internal grouping of data by experiment, subject, and condition. MOBBED has direct support for EEGLAB EEG structures, with plans to develop a database browsing function and a plugin to load and store to a MOBBED database. No direct support for Brainstorm is planned, but Brainstorm representations could be mapped to a MOBBED database.

EPILAB (Teixeira et al., 2011), another MATLAB-based analysis toolbox for EEG in the context of seizure prediction, creates a mapping of data files on a local disk so that multiple files can be treated as a single dataset for analysis. Similarly, MoBILAB (MoBILAB-homepage), a MATLAB toolkit under development at the Swartz Center for Computational Neuroscience at UCSD, uses memory mapped files to represent collections of related stream data including EEG, eye-tracking, video, audio, and motion capture. Direct support for mapping MoBILAB files to a MOBBED database is planned in the next release.

BCILAB (Delorme et al., 2011), a MATLAB toolbox for automating machine learning pipelines, has an internal caching system for holding and retrieving the results of expensive calculations during processing. BCILAB uses the internal caching system for cross-validation and motivated the transformations incorporated into MOBBED.

FieldTrip (Oostenveld et al., 2011) is another widely-used MATLAB toolbox for analysis of EEG and other electrophysiological data. FieldTrip is oriented toward the development of automated batch pipelines and presents a very clean scripting interface. Most FieldTrip functions have two types of input-output arguments: *data* and *cfg*. The configuration structure, *cfg*, contains the parameters specifying how the data is to be processed. The *data* structure is usually the result of a previous FieldTrip function call. This structure usually has a *cfg* subfield containing the FieldTrip function call that produced it. Researchers could adapt MOBBED to FieldTrip applications by storing both the *data* and *cfg* structures as SIMPLE datasets, replacing the .cfg field of *data* with the UUID of the stored *cfg* structure. To enable caching, researchers would need to add each FieldTrip function call to the TRANSFORMS table, replacing *data* and *cfg* structures by the UUIDs.

The PSOM pipeline system (Bellec et al., 2012) provides a lightweight scripting framework and execution engine for scientific workflows in MATLAB. The infrastructure manages jobs and functional “bricks” that are functions developed by the user. By using an appropriate naming convention, users could develop bricks that save and restore from a MOBBED database and incorporate them into the pipeline.

The MATLAB Database Toolbox (Mathworks) supports data cursors and queries in SQL from MATLAB. Several freely available MATLAB toolboxes (Almgren, 2005; Shvorob, 2007; Kerr, 2012) also support execution of SQL scripts from within MATLAB. Unlike these MATLAB SQL query toolkits, MOBBED is designed to provide SQL-free database access using a more familiar structure paradigm that can be easily incorporated into computational workflows.

## Discussion

Our primary goal in implementing MOBBED was to facilitate large-scale data-mining of event-rich time series, particularly those associated with realistic EEG. In a typical analysis of a laboratory experiment, a researcher might demonstrate a significant difference in an EEG feature such as the P300 or the power in a particular frequency band between epochs corresponding to different experimental conditions.

With access to a large MOBBED database, the researcher could then extract similarly tagged event conditions and determine whether a similar pattern was observed in other data collections. MOBBED directly supports retrieval of events within a window of events with specified tags or other characteristics. Assuming that the database datasets were richly-annotated with multiple event overlays, the researcher could ask which types of events were likely to co-occur. Supervised learning methods generally require labeling of the data at particular time points with “ground truth.” Tagged events can play the role of ground truth. Unsupervised learning techniques find patterns in the data or in data features. Often the most difficult step in unsupervised learning is determining what the data items in each group have in common, a process that can be facilitated by retrieving event information from a MOBBED database.

Another type of question that might be asked is how often particular features co-occur across data collections. A researcher could store these feature vectors as MOBBED data definitions and use the underlying capabilities of PostgreSQL for searching. PostgreSQL is a widely-used open source object-relational database that supports a variety of data types and extensions such as elements that are vectors and JSON-like unstructured query support. As of version 9.1, PostgreSQL supports K-nearest-neighbor indexing and some machine learning algorithms available through MADlib (Hellerstein et al., 2012). A user may compute feature vectors for a dataset, store them as part of the dataset, and retrieve records corresponding to these features or close to these features.

An alternative to the relational approach is the NoSQL approach as implemented by databases such as Cassandra (Hewitt, 2010) or MongoDB (Chodorow and Dirolf, 2010). NoSQL databases generally provide tables with unstructured rows that can be queried using *name:value* queries. These databases are highly scalable, but often don't have consistency guarantees and can be tricky to set up and tune, especially if they are distributed over multiple nodes. We chose a more traditional route because we were able to map the semi-structured problem space to a relational representation that is sufficiently flexible to implement the core use cases. We do not envision a computational scale that would exceed the capabilities of PostgreSQL systems. We felt the stability, power, and flexibility of PostgreSQL made it a logical choice.

The potential and need for individualized computational databases is just beginning to be realized. By providing a simple, turnkey, generalized database for MATLAB programmers, MOBBED can facilitate sharing and reuse of data. Although MOBBED can be used as a permanent archival database for a variety of data types, its primary focus is sharing and reuse of intermediate computations and standardized pipeline results among members of a group. MOBBED can facilitate the handling of large-scale analysis, including extraction of complicated event scenarios, searching for data based on features, and combining results across multiple studies that have been tagged using a common framework.

The MOBBED architecture is designed to accommodate very general types of data and currently supports three modalities: EEG (the default), SIMPLE, and GENERIC. The EEG modality assumes the data is in an EEGLAB EEG structure. It stores the entire structure as a large binary object, but fully explodes the events and their attributes as well as channel information and other attributes for searching and manipulation. The SIMPLE modality simply stores the dataset as a large binary object and does not explode events. Users use this modality for simple archiving of datasets and are free to assign additional tags and attributes for special purpose searching. The GENERIC modality allows the flexible creation of datasets that have exploded events and metadata. Users who wish to create a new modality called XXX simply identify the modality by adding an entry to the modality table using putdb and implement a MATLAB class called XXX_Modality that has a static save method. The implementations call the JAVA API using the underlying database transaction mechanism.

MOBBED has flexible data representations that can represent a variety of different types of data. It is also possible to use MOBBED in a cloud computing environment with computational workers running on the remote infrastructure and delivering results to local clients. MOBBED has the infrastructure to support common tagging of event types across multiple datasets to facilitate combining of data across studies using CTAGGER and related tools. The current release of MOBBED has limited support for stored procedures and nearest neighbor queries. User-friendly support for these features is planned for the next release, with the goal of enhancing capabilities for large-scale data mining. Also planned are plugins for direct integration into EEGLAB and the addition of direct support for MoBILAB representations.

MOBBED consists of a MATLAB library and an independent Java layer that manages the database connections using JDBC and performs operations as SQL queries. One could write an adaptor to store and retrieve data using other platforms and languages through this Java layer. MOBBED runs on MATLAB versions R2010b and higher and requires no additional toolboxes. The project is open source and is freely available at: http://visual.cs.utsa.edu/mobbed. MATLAB users do not need to download the source for the Java layer, as the appropriate Java archive files are included with the distribution. Source and issue reports for MOBBED are maintained at http://vislab.github.com/MobbedMatlab/

### Conflict of interest statement

The authors declare that the research was conducted in the absence of any commercial or financial relationships that could be construed as a potential conflict of interest.
